# Effects of Mental Training Through Imagery on the Competitive Anxiety of Adolescent Tennis Players Fasting During Ramadan: A Randomized, Controlled Experimental Study

**DOI:** 10.3389/fnut.2021.713296

**Published:** 2021-11-12

**Authors:** Sofien Fekih, Mohamed Sami Zguira, Abdessalem Koubaa, Anouar Bettaieb, Jamel Hajji, Nicola Luigi Bragazzi, Mohamed Jarraya

**Affiliations:** ^1^Higher Institute of Sport and Physical Education of Gafsa, Gafsa, Tunisia; ^2^Institute of Sport and Physical Education of Ksar Said, University of Manouba, Manouba, Tunisia; ^3^Higher Institute of Sport and Physical Education of Sfax, University of Sfax, Sfax, Tunisia; ^4^Department of Physiology and Lung Function Testing, Faculty of Medicine Ibn-El-Jazzar, University of Sousse, Sousse, Tunisia; ^5^Laboratory of Pharmacology, Faculty of Medicine of Sfax, University of Sfax, Sfax, Tunisia; ^6^Higher Institute of Applied Studies in Humanity of Mahdia, University of Monastir, Mahdia, Tunisia; ^7^Postgraduate School of Public Health, Department of Health Sciences (DISSAL), Genoa University, Genoa, Italy; ^8^Department of Neuroscience, Rehabilitation, Ophthalmology, Genetics, Maternal and Child Health (DINOGMI), Section of Psychiatry, Genoa University, Genoa, Italy; ^9^Laboratory for Industrial and Applied Mathematics (LIAM), Department of Mathematics and Statistics, York University, Toronto, ON, Canada

**Keywords:** Ramadan fasting, tennis, mental imagery training, cognitive and somatic anxiety, self-confidence, intensity, direction, sport psychology

## Abstract

This study aimed to analyze the effects of mental training through imagery on the competitive anxiety of adolescent tennis players fasting during Ramadan. This is an experimental study conducted with 38 male tennis players, randomly allocated to two groups: an experimental group (EG, *n* = 18), aged 16.9 ± 0.6 years, and a control group (CG, *n* = 20), aged 16.7 ± 0.8 years. The study was designed as a randomized, controlled experimental trial (registration code PACTR 202006847771700). CG watched historical videos of the Olympics, while EG performed mental training. The competitive anxiety state assessment was recorded four times. The first measurement was carried out 1 week before Ramadan, the second measurement during the first week of the month, the third measurement at the end of the second week, and, finally, the fourth measurement during the fourth week of Ramadan. Our results revealed a significant interaction (time × groups) for all competitive anxiety subscales. Higher intensity and direction scores for the cognitive and somatic anxiety subscales during Ramadan compared with before Ramadan for both groups could be reported at *P* < 0.001. Higher intensity and direction scores for the cognitive and somatic anxiety subscales during Ramadan compared with pre-Ramadan for both groups could be found at *P* < 0.01. This increase in scores was greater for the CG than for the EG in the middle and at the end of Ramadan at *P* < 0.001. Finally, for the self-confidence subscale score, results revealed that intensity and direction scores were lower during Ramadan compared with pre-Ramadan for the two groups at *P* < 0.01. The score for the intensity of self-confidence was higher for the EG compared with the CG at the end of Ramadan at *P* < 0.001. It was concluded that mental imagery training was effective in reducing anxiety (cognitive and somatic) and increasing self-confidence in the intensity dimension of adolescent tennis players who fast during Ramadan.

## Introduction

Anxiety is a complex, multidimensional construct that deals with the individual's disposition and response to stressors and also with the tendency to perceive and cope with stressful situations. One of the main research topics in the field of sports psychology is competition and precompetition anxiety, in terms of the key factors and underlying psychological mechanisms that influence it in sports environments (1). Despite a growing body of studies and empirical observations, athletes' state of anxiety and the way they face such a psychological state remain not fully elucidated and understood with regard to some particularities. These include, for example, the dimensions and the determinants of competition and precompetition anxiety as a psychological state in general or in specific situations, such as those of athletes who fast during the month of Ramadan. Indeed, this month is a period which appears to be difficult for all Muslims, and, in particular, for the majority of Muslim sportsmen who are willing to fast. This difficulty is reflected in the physical, mental, and psychological state of athletes (2–4), and, of course, this may have a profound impact on sports performance and related results/outcomes.

Some studies have tried to subjectively assess the general psychological state during Ramadan. The most commonly used tools were self-designed questionnaires. Most studies have shown that the scores obtained were higher during Ramadan compared with the control period. Indeed, Chtourou et al. (5) found that fatigue assessed by means of the “Profile of Mood States” (POMS) questionnaire was highest in the afternoon in 17-year-old footballers who were fasting during Ramadan. Leiper et al. (6) demonstrated that the ability to concentrate decreased in footballers who are fasting. Zerguini et al. (7) found an increase in the incidence of headaches and a decrease in mood and motivation during Ramadan among footballers. On the one hand, according to the literature, no study has been done on the state of anxiety of athletes during Ramadan. Regarding the relationship between sports discipline and anxiety levels, athletes competing in individual sports appeared to show a higher level of anxiety compared with athletes in team sports (1).

Tennis is considered as an individual sport which has certain peculiarities, which can result in competitive anxiety in athletes. For example, during a tennis match, the player carries out an intermittent activity, composed of high intensity interspersed with periods of active or passive recovery, on the physical level, that can be very hard on the psychological and emotional levels (8, 9). During a match, the player may experience a wide variety of emotions such as anger, fear, or negative thoughts. A number of mental qualities and skills are essential for a tennis player, such as emotional control, confidence, perseverance, and focus, among others. To the best of our knowledge, no study has been done on the state of anxiety for tennis players facing the consequences of fasting in the month of Ramadan. Nevertheless, stress, anxiety, and self-confidence were the elements most investigated under usual conditions. The most widely adopted theoretical model is the multidimensional theory of competitive anxiety (10), which subdivides competitive anxiety into two dimensions: cognitive and somatic. Some models also consider a third dimension called self-confidence. Studies have found a negative relationship between cognitive anxiety and athletic performance (11, 12). Likewise, the results of previous research have shown that somatic anxiety negatively affects the performance of competitive athletes (13, 14). In contrast, according to Millet et al. (12), increased self-confidence can enhance and maximize athlete performance outcomes. In this sense, it is important to identify conditioning/training strategies that can reduce anxiety (somatic and cognitive) and increase the self-confidence of athletes. According to Wang et al. (15), mental training can be an effective strategy to improve cognitive variables of athletes. Mental training refers to the creation of mental images from sensory processes stored in the memory and accessed without external stimuli (16). According to symbolic learning theory, a person is able to create a “mental sketch” that helps deal with a particular task (17). Brick et al. (18) stated that imagination can elicit the motor cortex and generate neuromuscular activation like performing a mental task. The same authors demonstrated that there are four major mental training techniques: motivation-specific, motivation-general, cognition-specific, and cognitive-general (18). The first two are used to improve motivation and emotional control ability, respectively. Specific- and general-cognitive mental training techniques are adopted by athletes to maximize the performance outcomes of a motor task or to resolve a situation that arises in competition, respectively. Regardless of the mental training technique adopted, studies have shown that mental training can be a good strategy for maximizing the performance of an athlete (19).

Even though some scientific findings have revealed that mental training improves the cognitive and/or physical performance of athletes (18, 19), it should be noted that none of these studies was specifically devised to examine the effect of mental training on competitive anxiety among tennis players during Ramadan. From a practical standpoint, this type of research may identify the effects of mental training on competitive anxiety in tennis players who fast during this month. In this sense, the results can be extremely important for the athletes and for the managers and coaches of this sport. In this context, the aim of the study was to analyze the impacts of mental training through imagery on the competitive anxiety of adolescent tennis players fasting during Ramadan. Even though mental training appears to be able to improve the control of an athlete, two hypotheses have been formulated: (a) Fasting in the month of Ramadan is expected to increase the scores for the intensity and direction of cognitive and somatic anxiety and decrease the scores of intensity and direction of self-confidence; (b) Training with mental imagery reduces the cognitive and somatic anxiety scores and increases the intensity and the self-confidence direction scores.

## Materials and Methods

The study protocol was reviewed in depth and fully approved by the “Ethical Committee for the Protection of Southern People” (C.P.P.SUD), Sfax, Tunisia (protocol reference C.P.P.SUD No. 0032/2017). The present study, a 4-week randomized controlled experimental study conducted during the month of Ramadan in 2017, was carried out based on the latest version of the Helsinki Declaration and its subsequent amendments.

The registration code for the trial is PACTR 202006847771700.

### Sample Size

*H*_0_ is the null hypothesis, which was formulated as *H*_0_: *m*_1_ = *m*_2_, while the alternative hypothesis is *H*_a_: *m*_1_ = *m*_2_ + *d*, where *d* is the difference between the two means. *N* = *n*_1_ + *n*_2_ is the total sample size, where *n*_1_ and *n*_2_ are the sample sizes for the experimental and control groups, respectively.

The total sample size was estimated using the following formula (1) (20):


$$N=\;\frac{(r+1)\cdot {\left(Z_{\frac{\alpha }{2}}+Z_{1-\beta }\right)}^{2}\cdot \sigma^{2}}{r\cdot d^{2}}$$

where *Z*_α_ is the normal deviate achieving statistical significance = 1.64 (5% level of significance), *Z*_1−β_ is the normal deviate at 1–β% power with β the % of type II error (0.84 at 80% statistical power), and *r* is calculated as the *n*_1_/*n*_2_ ratio (*r* = 0.67 gives the sample size distribution as 1:1.5 for two groups). Here σ and *d* are the pooled standard deviation (SD). These values were computed based on a similar hypothesis formulated in studies carried out in similar settings (21).

### Participants

The non-probabilistic sample is made up of 38 tennis players competing at the national level and participating in the Tunisian national tennis championship. To be included in the research, given the inclusion criteria utilized for other surveys with racquet sports athletes (15, 22), participants must: (a) have been tennis athletes for at least 2 years; (b) systematically be training for at least 6 h per week; and (c) register for the National Tennis Championship. They were recruited because they said they would be willing to fast for the entire month of Ramadan. These participants were randomly allocated to two groups: an experimental group (EG, *n* = 18), aged 16.9 ± 0.6 years old and a control group (CG, *n* = 20) aged 16.7 ± 0.8 years old. There were no significant differences for age (*P* = 0.34) and experience in the discipline (*P* = 0.28) between GE and GC before the survey (i.e., at the baseline). The anthropometric data for both groups (EG and CG) are presented in Table 1.

**Table 1:** Mean and standard deviation of descriptive research variables.

**Variables**	**Control group**	**Experimental group**	***P***
	**Mean (SD)**	**Mean (SD)**	
Height (m)	1.76 ± 0.1	1.77 ± 0.1	0.36
Body mass (kg)	66.2 ± 9.3	67.4 ± 5.9	0.32
BMI (kg/m^2^)	21.73 ± 0.87	22.02 ± 0.58	0.31

*SD, standard deviation; BMI, body mass index*.

### Procedure

This is a 4-week, randomized, controlled, experimental survey carried out during the month of Ramadan in 2017, recruiting adolescent male tennis athletes. Both groups (experimental and control) attended the same physical/technical training plan during Ramadan for 2 h per training session with a frequency of three times a week in the afternoon from 5 p.m. to 7 p.m.

The CG watched advertisement videos, whereas the EG underwent mental imagery training. Three mental imagery training sessions were performed per week, for a total of twelve sessions over a 4-week period during Ramadan. The sessions were held at 30-min intervals between the end of the mental imagery training session and the start of the technical/physical training session. No mental training session has been conducted without having completed physical/technical training. All mental imagery training sessions lasted approximately for 10 min in a quiet environment (near the tennis court), where the athletes wore the outfits they used to wear when competing (23). Videos of tennis players who were successful in competitions were used before each mental training session to aid the imagination of the athletes in the experimental group.

**Figure 1:**
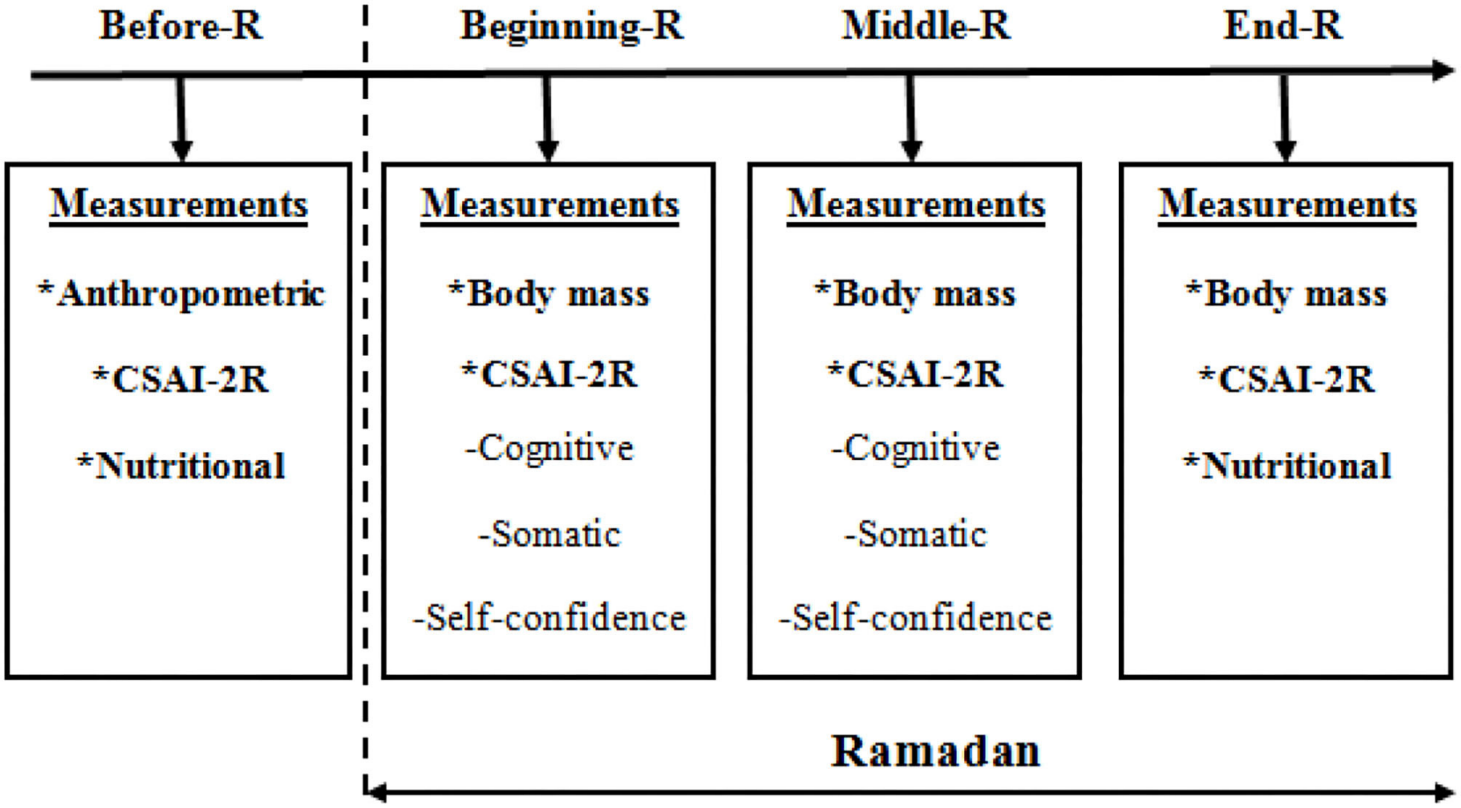
Experimental protocol adopted in the present study. CSAI-2R, The Tunisian version of the Competitive State Anxiety Inventory (24).

The recommendations of Brick (18) were used for the development and implementation of the mental training protocol. Therefore, the general type of cognitive imagination was adopted, asking athletes to imagine themselves being engaged in a competition. The following instructions were given to the athletes: (1) to create a mental situation using the first person; (2) to imagine the task at a speed close to reality; (3) to imagine positive situations during a competition; (4) to generate emotions (anxiety and mood) similar to those experienced during the competition. Two trainers expert in training in motor imagery were responsible for leading the interventions for the experimental and the control groups, with the principle of avoiding any bias between the groups. During this study, the assessment of dimensions of competitive anxiety was collected four times. The first measurement was carried out 1 week before Ramadan, the second measurement during the first week of the month, the third measurement at the end of the second week and, finally, the fourth measurement during the fourth week of Ramadan. To avoid any disturbance, the measurement points were recorded within 30 min before the commencement of the standard training. The evaluation of the anthropometric data was carried out a week before Ramadan, as pictorially represented in Figure 1.

### Measures

The food consumption record was taken by the participants over a period of three consecutive days using a food consumption log. Each participant was asked to note all the types of food ingested during three consecutive days. The average daily calorie intakes as well as the percentages of carbohydrates, fats, and proteins in the food were calculated (Table 2).

**Table 2:** Daily nutritional intake before and at the end of the fourth week of Ramadan.

**Variables**	**Before Ramadan**	**End Ramadan**
	**Mean (SD)**	**Mean (SD)**
Protein (%)	19.9 ± 2.6	20.2 ± 3.8
Lipids (%)	32.1 ± 5.81	30.6 ± 4.3
Carbohydrate (%)	50.4 ± 5.6	50.1 ± 3.6
Calorie intake (kcal/day)	3,147 ± 141.3	3,133 ± 182.2

*SD, standard deviation*.

To study the symptoms of competitive anxiety a few minutes before athletic competition, we used the Tunisian version of the revised competition anxiety state inventory (24). The CSAI-2R has been validated for Tunisian athletes and has demonstrated excellent psychometric properties (24). This tool allows the measurement of three subscales of competitive anxiety (cognitive, somatic, and self-confidence) which comprises of 16 items on a Likert scale. Each subscale includes three dimensions measuring intensity [from 1 (not at all) to 4 (a lot)], direction [from −3 (very unfavorable) to +3, (very favorable)] and frequency [from 1 (not at all) to 7 (always)]. After obtaining the consent of the coaches and parents/legal guardians, the athletes were asked to answer the items of the Tunisian version of CSAI-2R, 1 h before the competition (25). This psychometric tool can be used multiple times during a competitive season because its reproducibility is well established (10). We did not opt to use the frequency dimension of the CSAI-2R because this dimension analyzes the extent of competitive anxiety at the time of its filling. In addition, also other investigations have not adopted the frequency dimension of CSAI-2R (1, 26). For this sample, the internal consistency in the CSAI-2R ranged from 0.73 to 0.91 on the intensity scale and from 0.84 to 0.89 on the direction scale as identified (assessed by Cronbach's alpha) for the cognitive anxiety, somatic anxiety, and self-confidence subscales.

Measurements of height, body mass, and body mass index (BMI) were made using a board and electronic scale (Tanita, Tokyo, Japan).

### Statistical Analyses

Before proceeding to statistical analyzes, normality (Shapiro-Wilk test) and homogeneity of variance (Mauchly's sphericity test) were checked. For this sample, the internal consistency of intensity and direction dimensions was verified (assessed by Cronbach's alpha) for the cognitive anxiety, somatic anxiety, and self-confidence subscales. Then, the scores obtained from the responses to the different items of the competitive anxiety questionnaire were analyzed by means of the analysis of variance (ANOVA) on repeated measures [period (before Ramadan/start, middle, and end of Ramadan) × imagery (without/with)]. When the latter test showed a significant effect, the Wilcoxon test was utilized. If there was a significant effect, the Fisher's least significant difference (LSD) *post-hoc* test was used to compare the pairwise means. All observed differences were considered statistically significant for a probability threshold <0.05 (*P* < 0.05).

## Results

The descriptive data are reported in Table 1. No significant differences between the experimental and control groups before starting the intervention could be found, indicating the homogeneity of the two groups. Table 2 shows the means (±SD) of the daily calorie intake and the percentages of carbohydrates, fats, and proteins contained in consumed foods, recorded before, during, and the end of Ramadan. No significant differences could be identified for daily nutritional intake during the two periods.

Table 3 presents the means (±SD) of body masses and BMI of the two groups (experimental and control) at different times of the experiment (before, at the beginning, at the middle and at the end of Ramadan). Significant differences were observed for body mass and BMI, in the middle and at the end of Ramadan compared with before Ramadan for both the groups.

**Table 3:** Averages (± SD) of body mass and BMI recorded before and during Ramadan.

**Parameters**	**Groups**	**Before Ramadan**	**Beginning of Ramadan**	**Middle of Ramadan**	**End of Ramadan**	**Ramadan** × **Group interaction**		
			**Mean (±SD)**			***F*** **_(3.108)_**	***P***	**η_p_^2^**
Body mass (kg)	CG	66.2 ± 9.3	65.9 ± 8.7	64.2 ± 7.9^*^	63.8 ± 9.1^*^	5.99	0.017	0.08
	EG	67.4 ± 5.9	67.2 ± 5.2	65.3 ± 4.9^*^	65.1 ± 5.3^*^			
BMI (kg/m^2^)	CG	21.73 ± 0.87	21.58 ± 0.94	21.47 ± 0.93^*^	21.33 ± 0.94^*^	5.44	0.023	0.08
	EG	22.02 ± 0.58	21.91 ± 0.59	21.80 ± 0.57^*^	21.68 ± 0.57^*^			

*SD, standard deviation; EG, experimental group; CG, control group; BMI, body mass index*.

* *Significantly different from Bef-R at P < 0.05*.

Statistical analysis showed a significant interaction between Ramadan period (before/at the beginning/in the middle/at the end) × body mass [*F*_(3.108)_ = 5.99; *P* = 0.017]. A similar trend could be observed for BMI [*F*_(3.108)_ = 5.44; *P* = 0.023].

Regarding cognitive anxiety, the ANOVA results showed a significant interaction of the Ramadan period (before/at the beginning/in the middle/at the end) × groups for the dimensions of cognitive anxiety [intensity: *F*_(3.108)_ = 25.23, *P* = 0.001; direction: *F*_(3.108)_ = 2.73, *P* = 0.048] (Table 4). Comparison analyzes for the periods of Ramadan vs. before Ramadan revealed that the intensity and direction of cognitive anxiety during Ramadan were higher compared with before Ramadan for both the groups (all, *P* < 0.001). Regarding the differences between groups in the same time periods, the means of intensity dimension scores were higher for CG compared with EG in the middle and the end of Ramadan (*P* < 0.001).

**Table 4:** Variation in the dimensions of cognitive anxiety recorded before and during Ramadan.

**Dimension**	**Groups**	**Before Ramadan**	**Beginning of Ramadan**	**Middle of Ramadan**	**End of Ramadan**
				**Mean (±SD)**	
Intensity	CG	2.0 ± 0.2	2.7 ± 0.3^**^	3.4 ± 0.2^**^	3.5 ± 0.1^**^
	EG	2.2 ± 0.2	2.6 ± 0.2^**^	2.9 ± 0.2^^**^##^	2.8 ± 0.2^^**^##^
Direction	CG	−1.8 ± 0.5	−2 ± 0.5^**^	−2.3 ± 0.4^**^	−2.5 ± 0.3^**^
	EG	−1.6 ± 0.4	−1.8 ± 0.4^*^	−2.1 ± 0.3^**^	−2.4 ± 0.2^**^

*SD, standard deviation; EG, experimental group; CG, control group*.

*, ** *Significant difference from before Ramadan respectively at P < 0.05 and at P < 0.001*.

## *Significant difference from GC, at P < 0.001*.

Regarding somatic anxiety, the ANOVA results showed a significant interaction of the Ramadan period (before/at the beginning/in the middle/at the end) × groups for the dimensions of somatic anxiety (intensity: *F*_(3.108)_ = 23.9, *P* = 0.001; direction: *F*_(3.108)_ = 5.01, *P* = 0.003) (Table 5). Comparison analyzes for the periods of Ramadan vs. before Ramadan revealed that the intensity and direction of somatic anxiety during Ramadan were higher compared with before Ramadan for both groups respectively at *P* < 0.001 and *P* < 0.003. Regarding the differences between the groups in the same time periods, the means of intensity dimension scores were higher for CG compared with EG in the middle and the end of Ramadan (*P* < 0.001).

**Table 5:** Variation in the dimensions of somatic anxiety recorded before and during Ramadan.

**Dimension**	**Groups**	**Bef-R**	**Beg-R**	**Mid-R**	**End-R**
				**Mean (±SD)**	
Intensity	CG	1.6 ± 0.2	2.3 ± 0.2^**^	3.1 ± 0.3^**^	3.3 ± 0.3^**^
	EG	1.6 ± 0.3	2.2 ± 0.2^**^	2.6 ± 0.3^^**^##^	2.7 ± 0.3^^**^##^
Direction	CG	−0.9 ± 0.4	−1.3 ± 0.3^**^	−1.8 ± 0.3^**^	−2.3 ± 0.3^**^
	EG	−0.7 ± 0.3	−1.3 ± 0.3^**^	−1.9 ± 0.3^**^	−2.2 ± 0.3^**^

*SD, standard deviation; EG, experimental group; CG, control group*.

** *Significant difference from before Ramadan at P < 0.001*.

## *Significant difference from GC, at P <0.001*.

Regarding self-confidence, the ANOVA results showed a significant interaction of the Ramadan period (before/at the beginning/in the middle/at the end) × groups for the dimensions of self-confidence [Intensity: *F*_(3.108)_ = 8.75, *P* = 0.001; direction: *F*_(3.108)_ = 2.69, *P* = 0.05] (Table 6). Comparison analyzes for the periods of Ramadan vs. before Ramadan revealed that the intensity and direction of self-confidence during Ramadan were lower compared with before Ramadan for both groups respectively at (*P* <0.001 and *P* <0.05). Regarding the differences between groups in the same time periods, the means of intensity dimension scores were higher for EG compared with CG at the end Ramadan (*P* < 0.001).

**Table 6:** Variation in the dimensions of self-confidence recorded before and during Ramadan.

**Dimension**	**Groups**	**Bef-R**	**Beg-R**	**Mid-R**	**End-R**
				**Mean (±SD)**	
Intensity	CG	2.6 ± 0.4	2.2 ± 0.3^**^	1.9 ± 0.3^**^	1.6 ± 0.2^**^
	EG	2.3 ± 0.2	2 ± 0.2^**^	2 ± 0.1^**^	1.9 ± 0.2^**##^
Direction	CG	1.9 ± 0.3	1.4 ± 0.2^**^	1.3 ± 0.2^**^	1.1 ± 0.2^**^
	EG	2.2 ± 0.3	1.6 ± 0.4^**^	1.4 ± 0.4^**^	1.1 ± 0.4^**^

*SD, standard deviation; EG, experimental group; CG, control group*.

** *Significant difference from before Ramadan at P <0.001*.

## *Significant difference from GC, at P <0.001*.

## Discussion

The aim of this study was to analyze the effects of mental imagery training on the competitive anxiety of adolescent tennis players who fast during Ramadan. Two hypotheses have been formulated. First, fasting in the month of Ramadan increases the scores for the intensity and direction of cognitive and somatic anxiety and decreases the scores of intensity and direction of self-confidence. Second, training with mental imagery reduces the cognitive and somatic anxiety scores and increases the intensity score and the self-confidence direction.

### Effects of Fasting During Ramadan on Variation in Competitive Anxiety State

The results of this research indicated an observable increase in the dimensions of intensity and direction of cognitive and somatic anxiety for the CG during the fasting of Ramadan. Moreover, a decrease in the dimensions of intensity and direction of self-confidence indicates a reasonable probability that this conclusion may be true for tennis players who fast during Ramadan and who have similar characteristics reported in the present study. The state of anxiety of athletes and the way they deal with this psychological state warrant research with regard to a few particularities, for example, the state of competitive anxiety for athletes who fast during the month of Ramadan, which is relatively overlooked in the currently available scholarly literature. Indeed, a few studies have tried to subjectively assess the general psychological state during Ramadan. Most of these studies have shown that the scores obtained are higher during Ramadan compared with the control period. Moreover, Chtourou (5) found that fatigue, assessed by the POMS, questionnaire was highest in the afternoon in 17-year-old footballers fasting during Ramadan. A study by Leiper (6) found that the ability to concentrate decreased in fasting footballers. Zerguini (7) also reported an increase in the incidence of headaches and a decrease in the mood and motivation during Ramadan among footballers. On the other hand, according to the literature, no study has been done on competitive anxiety state for athletes who fast during Ramadan. Regarding the state of competitive anxiety measured outside the period of Ramadan, we can see that a tennis player is involved in an intermittent activity, consisting of great intensity efforts interspersed with active or passive recovery periods, which can be very hard on the physical, psychological, and emotional level (8, 9). For this, a certain number of mental qualities are essential for the tennis player to be successful, such as emotional control, confidence, perseverance, and concentration, among others. According to Di Rienzo and Fernandes (26, 27), high cognitive anxiety on the eve of a competition leads to increased muscle tension which, in turn, can lead to increased muscle stress, resulting in decreased performance and anaerobic resistance. Moreover, recently Fekih et al. (28) have shown a decrease in tennis service performance outcomes in terms of accuracy and stroke speed for tennis players fasting during Ramadan.

An increase in cognitive and somatic anxiety scores (in terms of intensity and direction), and also a decrease in self-confidence due to competitive anxiety during Ramadan fasting may be associated with the change in lifestyles and daily habits during this particular period. More specifically, one of these effects may be associated with the change in the sleep–wake cycle. In this context, the necessary duration of sleep of our players has been reduced compared with the shift in the times of food intake during Ramadan. Waterhouse (29) demonstrated that Muslims during Ramadan continue to eat and drink until late at night, which is likely to prevent them falling asleep. Then, they wake up for the last meal before dawn. These disturbances could reduce the duration of nighttime sleep. The fatigue, induced by this partial sleep deprivation, could explain the increase in cognitive and somatic anxiety scores in the dimensions of intensity and direction during Ramadan. On the other hand, dehydration may also be one of the factors responsible for the increase in cognitive and somatic anxiety scores during Ramadan. Indeed, the fasting of Ramadan is associated with a reduction of fluid, especially if carried out in a hot environment, which can strengthen the onset of dehydration. Based on our results, we found a decrease in body mass at the end of Ramadan compared with before Ramadan. This reduction could be related to a loss of body water, which is in line with the study by Sweileh (30). The negative effects of hypo-hydration on athletic performance outcomes are well documented in the literature (31). The increase seen in intensity and direction for cognitive and somatic anxiety during Ramadan is probably not related to changes in calorie intake, as we have not observed any difference in calorie intake and the percentages of lipids, carbohydrates, and proteins contained in food consumed during Ramadan compared with before Ramadan. This is in agreement with previously published studies (28, 32), which failed to observe any difference in the level of nutritional intakes during Ramadan compared with before Ramadan.

Finally, the increase in competitive anxiety during Ramadan can be attributed to a decrease in the arousal and motivation of participants (29, 33). Additionally, lower mood has been suggested as a factor responsible for lower performance outcomes during Ramadan, as a result of lowered self-confidence (34, 35).

### Effects of Mental Imagery Training on Changes in Competitive Anxiety State During Ramadan Fasting

The results of this research indicated a slight stabilization in intensity for the three subscales of competitive anxiety for the EG in the middle and at the end of Ramadan, which was not observed in the CG. In this sense, it seems that 30 min of training with the technique of mental imagery per week (three sessions of 10 min separated by 48 h during the month of Ramadan) can modify the dimension of intensity for the three cognitive anxiety subscales. According to Fernandes (1), cognitive anxiety is responsible for an impairment in decision-making related processes and concentration. Thus, given that tennis matches require a good stimulation strategy (8), which in turn requires maximum concentration, the high magnitude of cognitive anxiety could, maybe, generate early fatigue in tennis players due to poor preparation of the stimulation. Training with mental imagery, may, in a way, be effective in reducing the effect of fasting and stabilizing the intensity dimension for the three subscales of cognitive anxiety in fasting tennis players during Ramadan. This finding is in agreement with the results found by another work (36).

Regarding the somatic anxiety dimension, the results of this study showed a slight stabilization in the intensity dimension in the middle and at the end of Ramadan for the EG. This stabilization was not observed in the CG. In addition, a significant difference was observed between the two groups in favor of the EG in the middle and at the end of Ramadan. Based on a literature review, somatic anxiety can increase cardiovascular and neuromuscular stress (12). Thus, the increase in organic stress just before competition is linked to a decrease in cognitive and muscular performance outcomes (14). As such, somatic anxiety can lead to a decrease in the performance of tennis players during Ramadan. Thus, indirectly, training with mental imagery during the Ramadan fast can be adopted as a strategy of stabilizing the intensity dimension for somatic anxiety, thereby stabilizing the performance outcomes of adolescent tennis players also. According to Patel (14), the increase in somatic anxiety is linked to increased psychophysiological fatigue. For this, stabilization of or reduction in somatic anxiety resulting from mental imagery training appears to be essential to inhibit/counteract the early fatigue of tennis players, which can result in improved performance during competitions. Regarding self-confidence, results also showed stabilization in intensity for the EG in the middle and the end of Ramadan. This stabilization was not observed for the CG. The comparison between CG and EG by means of *post-hoc* tests suggests that the training with mental imagery may delimit the effects of the fasting by stabilizing the intensity dimension of the subscale of self-confidence in our tennis players. From this perspective, studies have shown that increased self-confidence can have a positive effect on athlete performance outcomes (26). Therefore, considering our results, training with mental imagery combined with physical and technical training during Ramadan may be an effective strategy to stabilize the dimension of intensity for the subscale of the self-confidence and, therefore, stabilize tennis performance.

Thus, coaches need to know their athletes to identify the magnitude required to increase self-confidence to maximize performance during training or competition in the month of Ramadan. It should be noted that confident tennis athletes often performed well in sports compared with athletes with low levels of self-confidence (1). In addition, the results of some studies have shown a positive relationship between self-confidence and athletic performance (12, 14). Thus, constructing a mental situation in the first person, imagining the task at a speed close to reality and positive situations during a competition and generating emotions (anxiety and mood) similar to the competition seem to be an essential cognitive strategy for mitigating the effects of fasting during Ramadan, and therefore stabilizing the intensity of competitive self-confidence in tennis players. This can lead to stability or increased performance during tennis training or in competitions (28, 36).

### Strengths of the Study

The present study has some strengths, including its novelty, its methodological rigor (in terms of study design and implementation, and statistical analyses conducted), and the wealth of indicators and data collected at various time-points.

### Limitations of the Study

Although revealing interesting results, this study has some limitations that should be mentioned. Brain and electromyographic signals were not assessed during mental training sessions for both groups (EG and CG). The use of the questionnaire is also a limitation. According to Fortes (37), the use of Likert scale-based questionnaires in surveys with repeated measures can generate a learning effect. Also, the Hawthorne effect, which is a type of effect linked with behavioral reactivity when individuals are aware of being observed, cannot be ruled out. Further, biopsychological indicators such as cortisol and testosterone were not evaluated during the tests. Finally, the results of this study must be interpreted with caution.

## Conclusions

This study represents a first attempt to examine the effects of mental imagery training on changes in the dimensions of competitive anxiety during fasting during Ramadan. Results showed that fasting during Ramadan increases both dimensions (intensity and direction) of cognitive and somatic anxiety and decreases both dimensions (intensity and direction) of self-confidence. A training program with mental imagery after regular training sessions can only stabilize the intensity dimension for the three subscales of competitive anxiety (cognitive, somatic, and self-confidence).

## Data Availability Statement

The original contributions presented in the study are included in the article/supplementary material, further inquiries can be directed to the corresponding author.

## Ethics Statement

The study protocol was reviewed in depth and fully approved by the Ethical Committee for the Protection of Southern People (C.P.P.SUD), Sfax, Tunisia: protocol reference C.P.P.SUD No. 0032/2017. The patients/participants provided their written informed consent to participate in this study.

## Author Contributions

SF, MZ, NB, and MJ conceived the study and wrote the manuscript. AK, AB, and JH critically revised the manuscript. All authors contributed to the article and approved the submitted version.

## Conflict of Interest

The authors declare that the research was conducted in the absence of any commercial or financial relationships that could be construed as a potential conflict of interest. The reviewer KT declared a shared affiliation, with several of the authors SF, MZ, AK, AB, JH, and MJ to the handling editor at the time of the review.

## Publisher's Note

All claims expressed in this article are solely those of the authors and do not necessarily represent those of their affiliated organizations, or those of the publisher, the editors and the reviewers. Any product that may be evaluated in this article, or claim that may be made by its manufacturer, is not guaranteed or endorsed by the publisher.
